# Limited Cross Plant Movement and Non-Crop Preferences Reduce the Efficiency of Honey Bees as Pollinators of Hybrid Carrot Seed Crops

**DOI:** 10.3390/insects10020034

**Published:** 2019-01-23

**Authors:** Ann Gaffney, Björn Bohman, Stephen R. Quarrell, Philip H. Brown, Geoff R. Allen

**Affiliations:** 1 Tasmanian Institute of Agriculture, University of Tasmania, Churchill Ave, Hobart 7005, Australia; ann.gaffney@uni.sydney.edu.au (A.G.); stephen.quarrell@utas.edu.au (S.R.Q.); p.h.brown@cqu.edu.au (P.H.B.); geoff.allen@utas.edu.au (G.R.A.); 2 School of Molecular Sciences, The University of Western Australia, 35 Stirling Hwy, Perth 6009, Australia; 3 School of Health, Medical and Applied Sciences, Central Queensland University, Callemondah 4701, Australia

**Keywords:** crop carrot, hybrid, honey bee, pollination

## Abstract

Pollination rates in hybrid carrot crops remain limited after introduction of honey bee hives. In this study, honey bee foraging behaviour was observed in commercial hybrid carrot seed crops. Significantly more visits were made to male-fertile (MF) rather than cytoplasmically male-sterile (CMS) flowers. Pollen was collected from bees returning to a hive, to determine daily variation in pollen loads collected and to what level the bees were foraging for carrot pollen. Honey bees visited a wide range of alternative pollen sources and made relatively few visits to carrot plants throughout the period of flowering. Visitation rates to other individual floral sources fluctuated but visitation to carrot was consistently low. The underlying rate of carrot pollen visits among collecting trips was modelled and estimated to be as low as 1.4%, a likely cause of the limited success implementing honey bee hives in carrot crops.

## 1. Introduction

In commercial hybrid carrots, cross-pollination is required between a cytoplasmically male-sterile (CMS) line, which does not produce pollen, and an F1 male-fertile (MF) line that does produce pollen [1]. Hence, pollinator visits to the CMS line are for nectar only, with self-fertilisation of the seed-producing CMS line prevented by the absence of pollen on the CMS flowers. The crops grown are dominated by the CMS line with CMS:MF ratios of 2:1 to 4:1 common [2,3]. The MF cultivar is later removed after seed set and the seeds produced by the CMS cultivar are harvested for the commercial production of carrots [3]. Hybrid carrot crops are generally considered to require supplementary pollination by managed pollinators such as honey bees (*Apis mellifera* Linnaeus) [4]. Earlier studies investigating open-pollinated carrot seed crops indicate honey bees to be abundant and carry high pollen loads in carrot crops [5]. However, the reliability of honey bees as hybrid carrot crop pollinators appears to have diminished because of an increased variability between lines [1,6,7].

This variability in honey bee mediated pollination has led to an increased focus on the use of alternative pollinators including other Hymenoptera and some Diptera [8,9]. Despite enhanced pollination observed when alternative pollinators are used to supplement honey bee pollination services [10], the presence of other pollinator species can be unreliable between seasons or locations, with many species also carrying few pollen grains on their bodies [11,12]. As seed growers are able to hire honey bee hives to place within the carrot fields over the flowering period (December–January in Australia), managed *A. mellifera* remain the primary pollinators in hybrid carrots.

The placement of managed honey bee hives used for crop pollination is largely based on current understanding of honey bee foraging behaviours, including foraging range.

Indeed, whilst bees may forage much farther away, they tend to forage within 300 m of their hive in the presence of suitable floral resources, and it is therefore recommended that bee hives should be placed around crops so that this foraging distance is not exceeded [13]. Competition between hives that are grouped together encourages bees to forage farther within a crop and thus usual pollination practice is to cluster groups of honey bee hives together [14]. When foraging, whether for pollen or nectar, bees use olfactory and visual cues (such as colour) and typically stay within one particular flower type [15,16]. This constancy or ‘floral fidelity’ means that the pollen carried on the corbiculae of honey bees may only be of a single pollen type [17]. However, this may not always be the case [18,19], as a single floral resource may be in insufficient quantity to ensure adequate pollen is collected to maintain hive health [20].

In general, the availability of food is of paramount importance to insect colonies such as honey bee hives. A food source must first be located and then its position communicated to other foragers so that it can be maximally exploited [21]. Nectar and pollen collected from flowers form the basis of all food requirements of both adult and brood bees. Pollen provides protein, fat, vitamins and minerals, whilst nectar is a source of carbohydrates [22]. Sources of nectar and pollen are ephemeral, so the proportion of workers collecting nectar or pollen at any one time varies to suit the composition and number of adults in the hive (males and workers), brood levels, the amount of food resources stored within the hive [23] and the availability of forage and prevailing conditions [24]. It follows that the food source quality is an important factor for bee forage preferences.

When honey bee numbers are low and crops are nectar-poor, bees may be lured to other plants that produce greater quantities of nectar [21]. As the quality and quantity of nectar in hybrid carrots is often low [25], many weeds and other plant species near crops may have a greater relative attraction to honey bees. To improve pollination, it has been suggested that carrot seed crops should not be located near other competing crops or floral resources, but preferably near habitats that support alternative pollinators with potential to pollinate the carrot umbels [26]. 

In this study, we investigate possible causes to the observed low pollination rates of hybrid carrot seed crops despite the use of managed honey bee hives. We first examine honey bee foraging patterns among both nectar-collecting and pollen-collecting bees within hybrid carrot seed crops. Specifically, we examine visitation frequency and duration of honey bee visits on MF and CMS umbels. We then look at daily pollen collection activity of bees from hives placed adjacent to hybrid carrot seed crops in relation to climatic variables. Finally, we collect pollen balls from foraging bees returning to the hive and quantify carrot pollen content, relative to pollen collected from other flowering resources over the flowering lifetime of the hybrid carrot seed crop. By examining the pollen collected by honey bees in a hive adjacent to the crop, foraging patterns and any specific preferences for competing flowering plant species may be discerned.

## 2. Materials and Methods

### 2.1. Visits of A. mellifera to Male-Fertile and Cytoplasmically Male-Sterile Carrot cultivars

The four field trials were conducted at three sites: StrathAyr Turf Systems (42.755°S, 147.403°E), Pty. Ltd, Bejo Seeds Pty. Ltd. (42.704°S, 147.445°E) and the University Farm (42.797°S, 147.426°E), all located in the proximity of Hobart, Tasmania, Australia. All trials were conducted during January 2002–2005. Queen right Langstroth bee hives were placed adjacent to an experimental plots of hybrid CMS and MF carrot flowers at commercially recommended hive densities (six hives/Ha). Each hive consisted of one eight frame deep brood box and one eight frame ‘ideal’ honey super. Each lower brood box contained six frames with approximately 60% brood (egg–pupae) and two outer frames containing honey and pollen (total eight frames). Each hive contained approximately 30,000 bees derived from a cross between the European dark bee and the Italian bee. Full site descriptions and additional management information for each location are available in Supplementary Information.

#### 2.1.1. Visit Counts

In Trial 1 (StrathAyr Turf Systems Pty. Ltd), visitations to a CMS line (1A) and unrelated MF line (2B) were counted. These observations were conducted by a single observer, over 6 days, on 92 pairings of umbels that had at least one honey bee visit. Only those umbels with >40% receptive flowers were included in the random selection of umbels for observations of bee behaviour. The observer watched four umbels, two of each line, simultaneously for five minutes per observation. Prior to the commencement of each 5-min observation period, the number of bees already present on each of the selected umbels was recorded. No distinction was made between bees alighting for the first time or returning to an umbel after departing for any period. Each umbel was observed only once for the duration of the trial.

To control for any genetic differences between cultivars, genetically similar CMS and MF cultivars (3A and 3B, both lines carry the same nuclear genome) were compared in Trial 2 (Bejo Seeds Pty. Ltd). Two observers, over three days, monitored 13 pairings of umbels that had at least one honey bee visit. Each umbel was observed only once. As any disparity in *A. mellifera* visitation between CMS umbels and MF umbels may also be due to greater numbers of bees foraging for pollen rather than nectar, both the number of bees visiting an umbel and their individual foraging behaviour was recorded. Bees carrying pollen on their corbiculae were categorised as pollen-foragers and bees with no pollen on their corbiculae were categorised as nectar-foragers. Pollen collecting honey bees were also characterised by their swift movements across the umbels with their abdomen moving rapidly from side to side. Nectar-collecting bees were observed moving more slowly and stopping at flowers to extend their proboscides to the flower [27]. *A. mellifera* visits were also divided into bees visiting and staying, or landing and abandoning umbels in less than 5 s. Due to very low levels of honey bee visitation in this trial each umbel pairing was observed for 20 min. 

Umbel visitation data was analysed using 2 × 2 contingency table analysis with a Pearson chi-squared test, which compared the proportions of the two bee visitation behaviours recorded (stay or abandon) to the MF or CMS umbels/lines. Due to non-significance being attained during this analysis, post hoc power and sample size analyses were also conducted to determine the statistical power and sample sizes required to observe a significant result. These analyses were conducted using the Proc Freq and Proc Power functions in SAS v9.4 (SAS Institute Inc., Cary, NC, USA).

#### 2.1.2. Counts of Pollen in Relation to Flower Type

To examine the influence of bee foraging type (pollen versus nectar) and carrot flower type (CMS versus MF) on the amount of pollen carried by honey bees, in Trial 3 foragers from MF flowers (*n* = 45) and CMS (*n* = 77) flowers were collected within a carrot crop at the University Farm. Carrots were planted in single rows with the bees collected from one row of MF carrot umbels and three rows of CMS carrot umbels.

All 122 collected bees were stored in Eppendorf^®^ centrifuge tubes (Thermo Fisher Scientific, Australia) and on return to the laboratory, were stored at −20 °C prior to examination. The bees had their hind legs removed to exclude the bias from the pollen on their corbiculae and enable comparison of pollen on the rest of their body. 

Pollen loads were analysed using a modified method from [28]. Individual insects were placed in 1.5 mL Eppendorf tubes containing 50 µL of solidified glycerol gelatin (40 g of melted gelatin in 60 mL of glycerol diluted with 100 mL of deionised water). The storage tube from which the insects had been retrieved was flushed with 400 µL of xylene, which was subsequently added to the centrifuge tube containing the insect. A further 400 µL of xylene was added to the centrifuge tube. The tubes were agitated on a vortex mixer for 3 min to displace the pollen from the insect. The insects were then removed, and the tubes centrifuged at 15,000 rpm for 1 min, after which the xylene was decanted. The pollen-impregnated glycerol gelatin pellets were removed with a fine-hooked needle, placed on microscope slides, heated to melting, and covered with cover slips. Light pressure was applied to spread an even film of glycerol gelatin over the slide surface beneath the cover slip. After the slides had set they were examined under a light microscope at 100× magnification. Morphologies of the different pollen, isolated on honey bees during pollen analysis are available in Supplementary Information. Counts of the total number of carrot pollen grains were made from 10 randomly selected fields of view for each slide. The total number of pollen grains collected from each individual insect was calculated from the ratio of the slide area examined in 10 fields of view to the total area of the cover slip.

Following counts, pollen load on the bodies was divided into two categories due to over dispersion and clumping of data: either low (<10 pollen grains) or high (≥10 pollen grains). The pollen load was analysed in R^©^ version 2.9.1 (Copyright 2009 The R Foundation for Statistical Computing) using the Cochran–Mantel–Haenszel (CMH) procedure [29] using three-way (pollen load, bee type and flower type) tables. Tests were against the null hypothesis that the pooled odds-ratio is equal to 1 (i.e. there is no interaction between rows and columns) to explore whether bee type (nectar- or pollen-foragers) or flower type (MF or CMS) influenced pollen load.

#### 2.1.3. Collection and Analysis of Pollen from Foraging Bees Returning to the Hive

One queen right Langstroth bee hive was placed adjacent to an experimental plot of hybrid CMS and MF carrot flowers in Trial 4 (University Farm). The hive consisted of one eight frame deep brood box and one eight frame ‘ideal’ honey super. The lower brood box contained six frames with approximately 60% brood (egg–pupae) and two outer frames containing honey and pollen (total eight frames). The hive contained approximately 30,000 bees derived from a cross between the European dark bee and the Italian bee. A front-fitting pollen trap was attached to the hive entrance for a period of 24 hours twice per week across the two-month carrot flowering season (Table 1). Pollen was collected on 13 separate days. The pollen trap consisted of a perforated metal grid through which the bees squeeze to enter the hive. In doing so, the pollen balls are removed and fall through a screen into a collection drawer. For collection data, see Table 1. As continuous pollen trapping is somewhat controversial due to the potential of deleteriously restricting protein resources to the hive [30], the trap was removed on days pollen was not collected. The total weight of all pollen balls collected during each sampling was weighed to the nearest mg and an estimate of the number of pollen balls collected on each occasion was calculated by extrapolating the average weight of an individual pollen ball based on the weight of a 100-pollen ball sub-sample. 

To test if weather conditions related to differences in pollen collection, the relationship between pollen ball count on any one day and the weather parameters: daily temperature minima, daily temperature maxima, rainfall, pan evaporation and hours of sunshine, was analysed. All meteorological data was obtained from the Australian Government Bureau of Meteorology’s Hobart Airport weather station located at 42.834° S, 147.503° E; within 12 km of all field sites. Because of over dispersion of the pollen ball data (i.e., the true variance is greater than the mean) this data was analysed using a negative binomial generalised linear model in R^©^ version 2.9.1. After fitting the full model, terms (i.e., the abovementioned weather parameters) were dropped based on their significance in the model until the model of best fit was obtained.

To identify the number of pollen sources, a sub-sample of 60 pollen balls from each day’s trap collection (total 1500 pollen balls) were further examined. Pollen balls were cut in half and each half was mounted onto a microscope slide with a drop of liquid glycerol gelatine. Both halves of the pollen ball were then examined using a binocular microscope at 40× magnification to identify the pollen morphotype and to make sure that each pollen ball contained only a single pollen type. Mixed pollen balls were excluded and replaced by another ball from the same trap collection. A survey of local flowering sources was conducted to within a radius of 2.3 km of the crop site to help with identification of pollen, other than carrot pollen, in the pollen balls. 

### 2.2. Modelling Visits to Carrot Flowers

R^©^ version 2.9.1 (Copyright 2009 The R Foundation for Statistical Computing) was used to estimate the most likely rate of carrot pollen collection by the colony. The observed counts of carrot pollen during each sampling period were adjusted for the estimated total number of pollen balls collected during that period from the sub-sample of 60 balls used to determine the carrot pollen count. The simulation model used Markov-Chain Monte Carlo methods (MCMC), a computational technique which allows samples to be drawn from the posterior distribution arising from a Bayesian calculation. Details of the model are provided within Supplementary Information.

## 3. Results

### 3.1. Visits of *A. mellifera* to Male-Fertile and Cytoplasmically Male-Sterile Carrot Cultivars

**Figure 1:**
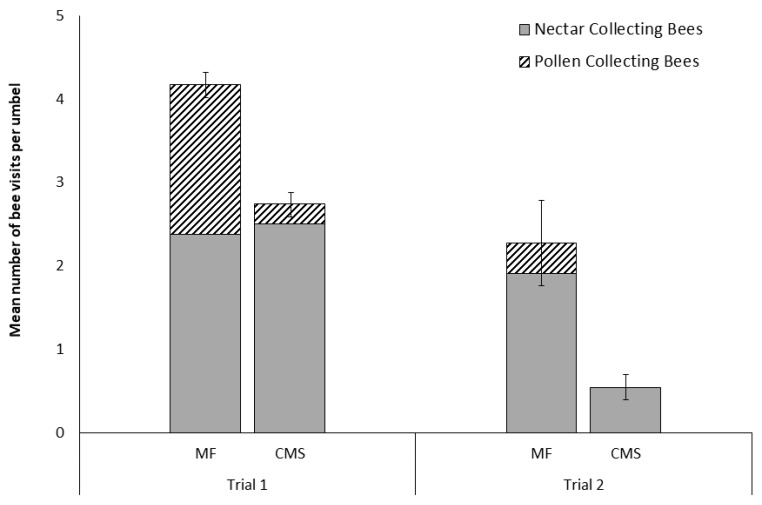
Number of *Apis mellifera* (mean ± SE) visiting pairs of male-fertile (MF) and cytoplasmically male-sterile (CMS) carrot umbels in trials. (Trial 1; n = 92 pairs, observations for five minutes per umbel. and Trial 2; n = 13 pairs, observations for 20 minutes per umbel) subdivided into proportions of nectar and pollen collecting bees observed.

In Trial 1, significantly more bees were observed foraging on the MF carrot lines (2B; *n* = 437) compared to the CMS carrot lines (*n* = 252; *t* = 5.16, d*f* = 91, *P* < 0.0001; Figure 1). This significant difference was driven by observed differences in the number of pollen foraging bees, with no difference observed in the abundance of nectar foraging bees between CMS and MF lines (*z* = −1.764, *P* = 0.078). Of these visitations, 37.8 % of the bees observed on MF umbels were pollen foraging compared to only 8.7 % of those observed on CMS lines. 

Very few bees (*n* = 31) were seen on carrot inflorescences in Trial 2 where the genetically similar MF and CMS lines (the same nuclear genotypes) were compared, suggesting foraging on other crops. Despite this, there was once again a significant difference between the visitation rates between the CMS and MF umbels (*t* = 5.04, d*f* = 12, *P* < 0.0005; Figure 1). In this case, 87 % of bee visits were to MF umbels and no pollen collecting bees visited CMS umbels. Nectar-collecting bees were observed visiting both MF and CMS umbels during both trials (Figure 1). 

During Trial 2, nectar-collecting bees stayed greater than 5 s in 67% of visits (*n* = 21) to MF umbels and in three of the only four visits made to CMS umbels. Similarly, pollen collecting bees stayed in 83% (*n* = 6) of visits to MF umbels. However, due to these low rates of bee visitation neither flower type (CMS or MF) nor bee type (pollen- or nectar-collector) significantly influenced visitation outcome (stay or abandon) (*χ*^2^ = 0.36, d*f* = 1, *P* = 0.849). Indeed, post hoc power analysis of Trial 2 data revealed a statistical power of just 0.70 for detecting a small effect with an estimate of 958 individual umbel observations (or 79.8 hours of observations) required to detect a significant difference if a similar standard deviation was observed in a replicated trial. 

**Figure 2:**
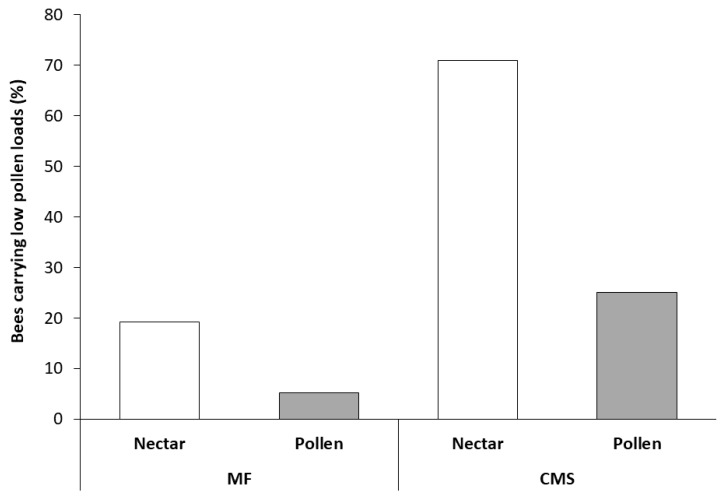
Percentage of *Apis mellifera* carrying low pollen loads (<10 pollen grains) visiting male-fertile (MF) and cytoplasmically male-sterile (CMS) carrot umbels. Both bee type (nectar- or pollen-foragers) and flower type (MF or CMS) significantly influenced bee body pollen count. The sample size was 122 bees in total. Samples per column are MF nectar = 69, MF pollen = 6, CMS nectar = 26, CMS pollen = 19.

In Trial 3, both bee type and flower type significantly influenced bee body pollen counts (Figure 2). A higher number of bees with low pollen loads was found, irrespective of bee type, on CMS flowers than MF flowers (*χ*^2^ = 14.88, d*f* = 1, *P* < 0.001), with low pollen counts six times more likely on CMS than MF flowers. As would be anticipated, more low pollen load counts were observed, irrespective of flower type, on nectar-collecting bees than pollen-collecting bees (*χ*^2^ = 12.76, d*f* = 1, *P* = < 0.001) with low pollen counts eight times more likely on nectar-collecting bees than pollen-collecting bees. Similarly, high pollen loads were observed on 95% pollen collecting bees (*n* = 18) collected from MF flowers and 5 of the 6 pollen collecting bees found on CMS flowers, all of which were carrying carrot pollen and therefore moved from the MF to CMS lines.

### 3.2. Collection and Analysis of Pollen from Foraging Bees Returning to the Hive 

The captured quantity of pollen returned to the hive averaged 2629 ± 1810 (S.D.) pollen balls per day, although this varied greatly between days, ranging from 690 to 4605 balls per day (Table 1; global mean = 1387 ± 1243 (S.D.)). The weight of a 100-ball sample of pollen averaged 0.72 ± 0.09 (S.D.) g per collection interval, but varied between 0.55 g and 0.87 g, suggesting that some pollen types weigh more than others, or alternatively are bound differently by the bees. There was no significant correlation between the 100-ball pollen weight and total pollen weight on that day (mean = 10.31 ± 0.09 (S.D.); Spearman *r* = 0.33, *n* = 23, *P* = 0.13). There was a significant difference in the total weight of pollen collected between mornings (mean = 14.95 ± 12.67 g S.D.) and afternoons (mean = 5.59 ± 3.32 g (S.D.)) (Wilcoxon signed ranks test, *Z* = −2.6, *n* = 10, *P* = 0.009). The weight of 100-ball samples of pollen did not significantly vary between mornings and afternoons (Wilcoxon signed ranks test, *Z* = −0.10, *n* = 10, *P* = 0.92). Only the minimum temperature (*z* = 3.71, *P* < 0.0001) and level of pan evaporation (*z* = −3.55, *P* < 0.0001) significantly influenced the count of pollen balls. A 1 mm increase in evaporation caused the expected pollen ball count to decrease by a factor of 0.79, holding minimum temperature constant, whilst a 1 °C increase in the minimum temperature caused the expected pollen ball count to increase by a factor of 1.20, holding evaporation constant.

The survey of the surrounding area revealed that there were few flowering plants immediate to the experimental carrot crop (Table 2). Some sparse weedy growth was present in and around the carrot plants. There was eucalypt dominant dry sclerophyll forest with various species of flowering plants between 1.9 km and 2.2 km from the experimental site with domestic gardens at 2.3 km. The intervening areas were devoted to farmland used for vineyards and orchards (not flowering at the time of the study). Honey bees would need to travel a minimum of 0.5 km into the surrounding countryside to access the larger pollen loads provided by flowering trees. Whilst there were few private gardens in the vicinity, these were observed to have a variety of exotic flowering plants. 

Thirty-eight different morphotypes of pollen were differentiated from the pollen balls examined, though not all pollen morphotypes could be attributed to plant taxa. Carrot pollen and 22 of the remaining 37 pollen morphotypes are illustrated in Supplementary Information. Very few pollen balls were of carrot pollen. Of the 1500 pollen balls examined, just 19 or 1.27 % were composed of carrot pollen. The underlying rate of carrot pollen visits made among collecting trips was estimated by the MCMC model to be just 1.4 % of collecting trips but varied between 1.3 to 2.2 % on any one day (see Supplementary Information). Twenty-two of the remaining 37 pollen types identified were found in lower numbers than carrot pollen, representing 10.3 % of the balls examined. 

Sampling of collected pollen balls revealed that bees tended to forage irregularly and the species of plants that bees foraged on varied from day to day (Table 1). From the samples counted, the number of plant species that the bees collected pollen from on any one day averaged 14.4 ± 0.7 (S.E.) plant species. Pollen types BA, AA (*Acacia*), HA, NA, WA (Malvacae) and QA (Myrtaceae) were each found in at least 100 pollen balls and represented 70% of the all pollen balls examined. Visitation to plants with these six pollen types varied across the season. Pollen type BA was the most frequently found on the first three collection days, representing 57%, 23% and 23% of the 60-pollen ball sub-sample on these dates but was subsequently present in low amounts, while AA (*Acacia*) became the dominant pollen in the middle of the sampling period, and type HA and NA were dominant at the end of the season. The six dominant pollen types measured between 30 µm and 50 µm in diameter. The other pollen morphotypes collected had a diameter of between 15 µm and 50 µm with the single exception of type QA (Myrtaceae) which had a relatively large diameter size of 80 µm ± 5 µm (S.E.). Carrot pollen measures 30 µm ± 5 µm (S.E.), which is within the range of pollen sizes of all morphotypes collected. 

## 4. Discussion

Honey bees from hives located adjacent to carrot seed crops displayed a preference for foraging in alternative species to carrot, with only a small percentage, estimated at 1.4%, of the pollen load on bees found to be carrot pollen. Of the bees visiting carrots, a preference for MF flowers, containing both pollen and nectar, was recorded. Between 63% and 87% of honey bee visits were to MF rather than CMS flowers, and nectar collecting bees were more likely to carry low pollen loads on their body than pollen collecting bees. As CMS flowers contain nectar but no pollen, only nectar collectors would be expected to visit these flowers. The likelihood of low pollen loads on nectar collecting bees visiting the CMS flowers, combined with the low frequency of visits to CMS in comparison to MF flowers in the crop, and a preference to visit alternative species to carrots, can help to explain the poor seed set observed in hybrid carrot seed crops despite the placement of hives near the crops [31].

It has previously been recommended that open-pollinated carrot seed crops should not be located near other crops which may provide competition for the attention of honey bees [26]. The results of our study extend the recommendation to hybrid carrot seed crops utilising CMS parent lines. Our results support similar findings by Galuszka and Tegrek [7], who also reported low pollination in carrot crops when alternative pollen and nectar sources were available. Similar crop management problems have been demonstrated in avocado crops which failed to rival competition from citrus flowers [32], alfalfa crops which were out-competed by roadside gumweed up to 1 mile (1.6 km) away [33] and of onions where between only 6% and 8% of pollen collected from returning bees was found to be onion pollen [34]. 

Several studies have established that bee activity is restricted by lower minimum temperatures [35,36]. As *A. mellifera* are heavily utilised as pollinators of carrot seed crops, the consequence of flowering over a period when morning temperatures are cool would most likely lead to lower seed set during these periods. Although daily maximum temperature alone was not a significant factor in relation to the amount of pollen collected by the honey bee colonies on any one day, the rate of evaporation, which tends to increase with increasing temperature, was. Furthermore, pan evaporation also depends on the temperature difference between the air and the evaporating surface, the relative humidity, solar radiation and wind speed, all factors that are known to impact on honey bee foraging activity [37,38]. 

The examination of pollen from the corbiculae of returning honey bees showed that *A. mellifera* from a single hive were prepared to visit a highly diverse range of other flowering plants. If there are one or more specific alternative plants that bees are visiting, seed growers may be able to manage their crops by manipulating the flowering time of their carrot crops to a time when competition is least likely, thereby reducing the pollination deficit. Alternatively, if there is low crop visitation of *A. mellifera* due to general competition from surrounding flora then crop site selection or removal of competitive flowering plants should be considered where possible [13,27,34]. However, this study suggests the removal of competing floral resources is not likely to be feasible due to costs or scale of the removal required. 

Honey bees showed a distinct preference for visiting MF carrot umbels rather than CMS carrot umbels as has been observed repeatedly elsewhere [7,9,12]. This lower visitation rate led to lower carrot pollen loads both on honey bees’ bodies and within pollen collected on the corbiculae. This finding may be due to honey bees preferentially foraging more frequently along rows, rather than across rows, which has been observed elsewhere [12]. Whilst *A. mellifera* were reoccurring visitors, they did not appear to be frequent visitors to the carrot crops relative to other flowering resources outside the crop, and even less frequent visitors to the CMS umbels compared to the MF umbels located within the crop. Therefore, it appears that the attractive floral attributes of carrot umbels successfully used by honey bees in open-pollinated seed crops may have been lost during the plant breeding process or that other repulsive attributes may have been accidentally bred into many hybrid carrots lines. Indeed, further work is required to elucidate the differences in quality and quantity of the floral cues and subsequent rewards provided to pollinators including honey bees. 

It is currently believed that bees are unable to identify and preferentially forage for pollen based on its protein content alone and therefore may not always collect pollen that infer greater nutritional benefit to the colony [39,40]. However, it does appear that bees are able to discern pollen with higher amino acid [41] and lipid [42] content and to learn pollen scents [40,43,44] and potentially use these cues when pollen foraging, there-by maintaining their floral fidelity [45]. More recently, some aspects for nectar quality and floral volatiles from hybrid carrot flowers were reported [46,47]. 

## 5. Conclusions

In conclusion, the results of our study, although performed over a limited number of fields, point to poor retention of bees foraging within the carrot fields studied. Similarly, the diversity of pollen collected from a single managed honey bee hive indicated an extremely low preference for the hybrid carrot pollen compared to the floral resources outside the crop. To better understand these findings we recommend further, replicated studies to determine the consistency across fields to better inform growers on how and where hives should be placed. Furthermore, replicated studies examining the variability in individual hive responses focused on hive nutritional or queen laying status may aid in the improvement of hybrid crop pollination. By extending future investigations to cover both nectar and pollen attributes in unattractive hybrid carrot, and attractive open-pollinated carrot cultivars, coupled with behavioural assays of these cues and rewards in isolation, a better understanding of the key factors behind the poor bee-attraction to hybrid carrot flowers should be ascertained. Once understood, these qualities could be included as traits desired by seed production companies during the development of new cultivars in the future.

## Figures and Tables

**Table 1:** Collection date, time, pollen ball data, weather conditions, and flowering and pollen yields on day of collection.

Collection Date	Time	Total Weight (g)	100-Ball Weight (g)	Est. Pollen Balls ^#^	Pollen Balls/day	T_min_ (°C)	T_max_ (°C)	Rain (mm)	Evaporation (mm)	Bright Sunshine (h)	Crop in Flower (%) ^⬧^	Carrot Pollen (%) *
**06 Jan**	12:00	7.81	0.67	1175	2431	7.4	25.5	0	5.8	12.6	32	1.7
**06 Jan**	17:30	10.04	0.80	1256								0
**12 Jan**	12:00	7.67	0.68	1125	1591	8.6	20.4	0	8.0	12.6	40	3.3
**12 Jan**	21:30	3.66	0.78	466								6.7
**15 Jan**	12:00	6.94	0.68	1016	1286	12.5	24.9	0	9.6	9.7	49	3.3
**15 Jan**	21:00	2.21	0.82	270								1.7
**19 Jan**	12:00	4.40	0.64	690	1112	5.1	24.8	0	4.8	13.5	58	1.7
**19 Jan**	21:00	2.91	0.69	422								1.7
**23 Jan**	12:00	6.38	0.55	1160	1819	11.4	22.2	0	8.0	11.3	59	3.3
**23 Jan**	21:00	3.85	0.58	660								3.3
**04 Feb**	12:00	33.75	0.78	4327	5953	11.4	22.3	0	3.2	10.2	76	0
**04 Feb**	21:45	10.19	0.63	1626								0
**10 Feb**	12:00	14.00	0.79	1770	2588	11.7	18.2	0	4.8	4	87	0
**10 Feb**	20:45	6.23	0.76	818								1.7
**13 Feb**	12:00	34.87	0.76	4605	5054	10.5	23.4	0	5.6	8.5	80	0
**13 Feb**	20:45	2.52	0.56	449								3.3
**16 Feb**	12:35	33.49	0.82	4109	5288	10	20.7	0	4.2	12.4	73	0
**16 Feb**	21:00	8.29	0.70	1180								0
**20 Feb**	21:00	5.57	0.79	702	702	10.3	24.9	2.8	5.8	7.1	62	0
**24 Feb**	21:30	8.36	0.79	1062	1062	4.1	18.8	0	5.4	11.3	52	0
**27 Feb**	12:00	12.56	0.76	1650	2656	13.9	23.3	0	5.8	6.9	57	0
**27 Feb**	20:00	8.79	0.87	1005								0
**01 Mar**	12:30	2.61	0.70	374	NA	9.5	17.7	1.4	2.2	4.4	60	0

^#^ Pollen ball numbers were estimated by weighing 100 balls from each sample. ^⬧^ Percentage of crop in full bloom was calculated by counting the number of receptive umbels in a 100-umbel sample. * Carrot pollen percentage indicates the number of pollen balls containing carrot pollen.

**Table 2:** Plant types observed in surrounding district to experimental plot. Infrequent <10 plants observed, frequent 10–50 plants observed, very frequent >50 plants observed.

Plant type	Family	Distance (km)	Frequency
***Chenopodium alba*, fat hen**	Chenopodiaceae	0	Frequent
***Hypochoeris radicata,* cats ear**	Asteraceae	0	Frequent
***Malva* sp., mallow**	Malvaceae	0	Frequent
***Trifolium* sp., clover**	Fabaceae	0	Frequent
***Plantago lanceolata*, plantain**	Plantaginaceae	0	Frequent
***Raphanus raphanistrum*, wild radish**	Brassicaceae	0	Frequent
***Taraxacum* sp., dandelion**	Asteraceae	0	Infrequent
***Gazania* sp.**	Asteraceae	0.5	Frequent
***Onopordum* sp., thistle**	Asteraceae	0.5	Infrequent
***Rosa rubiginosa*, sweet briar**	Rosaceae	0.5	very frequent
***Rosa* sp., rose**	Rosaceae	0.5	single plant
***Rubus fruticosus*, blackberry**	Rosaceae	0.5	single plant
***Bursaria spinosa*, prickly box**	Pittosporaceae	2.2	Frequent
***Cassinia* sp.**	Asteraceae	2.2	Frequent
***Eucalyptus* sp.**	Myrtaceae	2.2	Frequent
***Ozothamnus* sp.**	Asteraceae	2.2	Frequent
***Asteraceae* sp., daisy**	Asteraceae	2.7	Infrequent
***Lobularia maritimus*, sweet alyssum**	Brassicaceae	2.7	Infrequent
***Pelargonium* sp.**	Geraniaceae	2.7	Infrequent
***Tropaeolum majus*, nasturtium,**	Tropaeolaceae	2.7	Frequent
***Acacia mearnsii*, black wattle**	Fabaceae	0.5–2.3	Frequent

## Supplementary Materials

The following are available online at http://www.mdpi.com/2075-4450/10/2/34/s1, Field site descriptions, MCMC simulation model information, Pollen morphology information.
