# Supplementary material for: Limited Cross Plant Movement and Non-Crop Preferences Reduce the Efficiency of Honey Bees as Pollinators of Hybrid Carrot Seed Crops
Source: Insects. 2019 Jan 23;10(2):34. doi: 10.3390/insects10020034 (PMC6410176; doi:10.3390/insects10020034)
Supplement: Supplementary file 1 [file insects-10-00034-s001.pdf]

insects

MDPI

Supplementary information

Limited Cross Plant Movement and Non-crop Preferences Reduce the Efficiency of Honey Bees as Pollinators of Hybrid Carrot Seed Crops

Ann Gaffney [1], Björn Bohman [2*] Stephen R Quarrell, Philip H Brown[3] and Geoff R Allen [1]

[1]Tasmanian Institute of Agriculture, University of Tasmania, Churchill Ave, Hobart 7005, Australia;
[2]School of Molecular Sciences, The University of Western Australia, 35 Stirling Hwy, Perth 6009, Australia
[3]School of Health, Medical and Applied Sciences, Central Queensland University, Callemondah 4701, Australia

*Correspondence: bjorn.bohman@uwa.edu.au; Tel.: +61-8-6488-3515

1. Field site descriptions

Following preliminary trials in 2001/2002, experiments were conducted at three sites in southern Tasmania during three consecutive summer seasons from 2002/2003 to 2004/2005. The experimental sites were located at

i. Bejo Seeds Pty. Ltd., (42.704° S, 147.445° E)
ii. StrathAyr Turf Systems Pty. Ltd. (42.755° S, 147.403° E)
iii. University Farm, University of Tasmania (42.797° S, 147.426° E).

All sites were within 30 km of Hobart and within 15 km of each other (Figure).

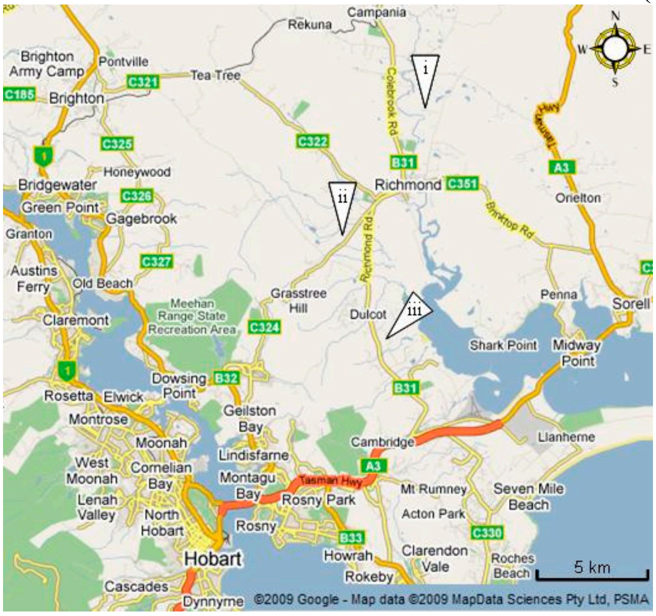

Figure S1. Location of trial sites. – i) Bejo Seeds Pty. Ltd, (42.704°S, 147.445°E), ii) StrathAyr Turf Systems Pty Ltd.(42.755°S, 147.403°E), iii) University Farm, University of Tasmania (42.797°S, 147.426°E)

Insect trapping and observations were conducted and umbel trimming was used at some sites to promote lateral flower stem development so that pollinator activity could be observed over an extended carrot growing season. The crop area of these sites and the activities conducted for each trial are detailed in Table S1. Traps were placed in trimming trials to take advantage of the extended carrot flowering time and thus the longer carrot and insect monitoring season. Hives were present adjacent to the carrot crop in all trials. Further details of these trials follow Table S1.

Table S1. Details of individual field trials.

Trial	Trial Date	Site	Total Planted Area (m^2)	Planted Area Used (m^2)	Hives Nearby	Cultivars Planted	Insecticide Spray Dominex®
1	Jan-03	StrathAyr	40000	288	> 1	PN6, MY1	Unknown
2	Dec-04/Jan-05	Bejo Seeds	1600	260	1	PF1, PBF1, MX1	None
3	Jan-05	Bejo Seeds	1600	260	1	PF1, PBF1, MX1	None
4	Dec-03/Jan-04	University Farm	1100	240	1	PN6 MY1	None

Carrot Cultivars

The carrot cultivars used in all field trials are listed in Table S2. Each cultivar has been given a unique experimental identification number.

Table S2. Cultivars of carrots used in field trials

Experimental ID	Root Type	CMS Type	Colour
PBF1	Berl/Flakee	Petaloid	Light Green
PF1	Flakee	Petaloid	White
PN6	Nantes	Petaloid	Light Green
MX1		Male-fertile	Light Green
MY1		Male-fertile	White

Field Trial Layouts

Field Trial 1 – January 2003 - StrathAyr Turf Systems Pty. Ltd. (288 m^2)

Carrots were planted according to the standard carrot planting layout detailed above. The planting layout was consistent with commercial carrot seed crop layout i.e. two beds of MF carrots (type MY1), six beds of CMS carrots (type PN6) then two beds of MF carrots (Type MY1). Only the CMS plants (PN6) were included in this experiment. A 60 m x 4.8 m section of this crop containing six beds of CMS carrots (cultivar PN6) was marked out and divided lengththwise into two blocks. Each of these blocks was then divided into six plots which were 5 m x 4.8 m each. Six different trimming treatments were randomly allocated to the plots within each of the two blocks. Each treatment plot was 1.8 m x 5 m (Figure S2).

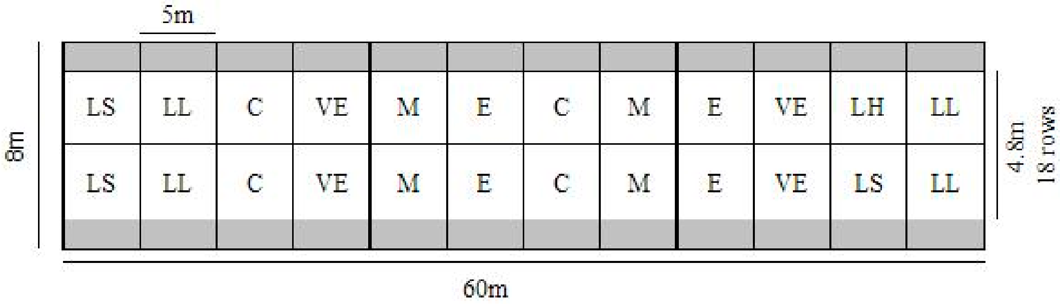

Figure S2. Field trial 1 – Trimming treatments; C=Control, VE=Very Early, E=Early, M=Mid, LL=Late Trim – Light, LS=Late Trim – Severe.

All of the carrot trimming treatments were conducted using a line trimmer. The *very early*, *early* and *mid* treatments were trimmed so that all vegetation 50 cm above ground level was removed. The first trimming treatment was conducted when 50 % of the carrots were at an extension of 30 cm or more. Trimming of the *late* trimming treatments were conducted at the same time, just prior to the opening of primary umbels. Carrot plants in the *late-severe* treatment were trimmed to 60 cm above ground level and carrot plants in the *late-light* treatment were trimmed to 75 cm above ground level. Trimming treatments and dates are listed in

Table S1.

Table S1. Trimming dates of carrots used in Field trial 1

Treatment Name	Trimming Date
Control	Not Trimmed
Very Early	14 October 2002
Early	30 October 2002
Mid	13 November 2002
Late - Severe	27 November 2002
Late - Light	27 November 2002

Field Trial 2 and 3 – December 2004/January 2005 – Bejo Seeds Pty. Ltd. (260 m^2)

This trial was a randomised block design. Eight blocks were divided into two plots of 2.4 x 5 m. Each plot was planted with one bed of an MF carrot (MX1) and then two beds of either carrot cultivar PF1 or PBF1. Each block contained a plot of PF1 and a plot of PBF1 (Figure S3).

Figure S3. Field Trial 2 and 3 crop layout

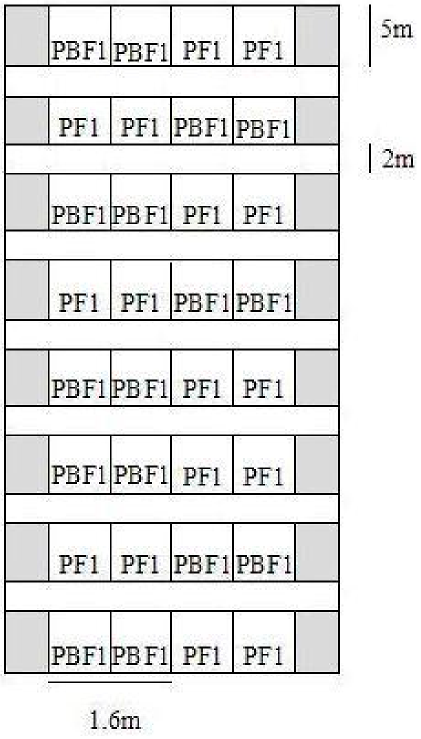

Field Trial 4 – December 2003/January 2004 - University Farm (240 m²)

The planting layout and carrot cultivar in this trial, MY1 and PN6, were the same as those used in Trial 1. Only treatments control, *early and mid, late-severe* and *late-light* were used. Twenty different treatment plots 2.4 m x 5 m were randomly allocated to a 50 m x 4.8 m block of CMS cultivar PN6. Each treatment plot was 2.4 m x 5 m (Figure S4). Trimming treatments and dates are listed in Table S4.

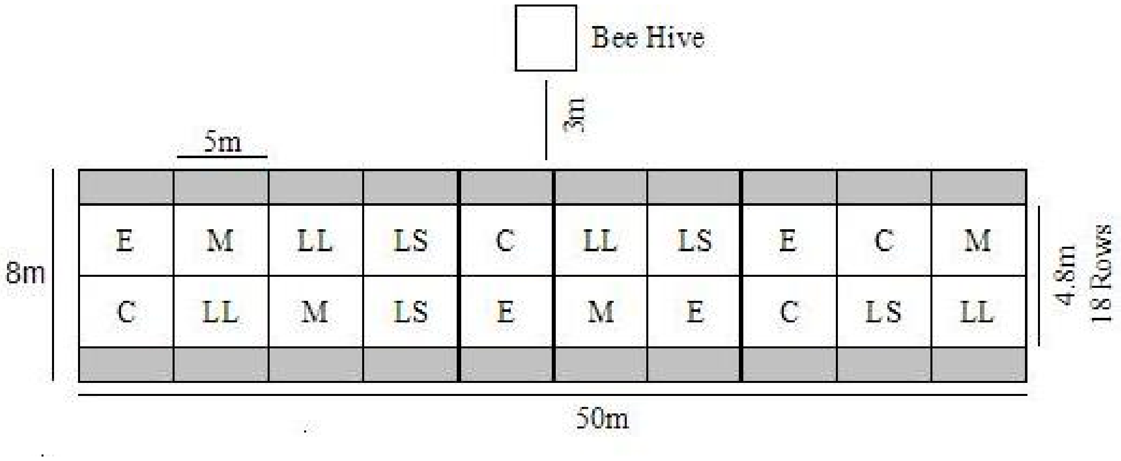

Figure S4. Field Trial 4 -Treatments; C=Control, E=Early, M=Mid, LL=Late Trim – Light, LS=Late Trim – Severe.

Table S4. Trimming dates of carrots used in Field trial 4

Treatment Name	Trimming Date
Control	Not Trimmed
Early	15 November 2003
Mid	1 December 2003
Late - Severe	23 December 2003
Late - Light	23 December 2003

2. MCMC Simulation model

Method

Markov Chain Monte Carlo (MCMC) is a computational technique which allows samples to be drawn from the posterior distribution arising from a Bayesian calculation.

Over the course of the last decade the accessibility and use of MCMC tools has increased substantially. The original BUGS (Bayesian analysis using the Gibbs Sampler) software has diversified into a family of tools which now includes WinBUGS (Lunn *et al*., 2000), OpenBUGS (Thomas *et al*.,2006), and JAGS (Just Another Gibbs Sampler) (Plummer, 2003); CODA (Plummer *et al*., 2009) has enabled a standardised format for post-processing

MCMC posterior samples; with all of these tools (and many others) accessible from within the R statistical environment (R Development Core Team, 2010), providing interoperability and ease of use for a wide (and growing) range of MCMC tools. Recent texts on the use of Bayesian MCMC methods in ecology include McCarthy (2007); Bolker (2008); Zuur *et al*. (2009).

We used R as an interface to JAGS to estimate the rate at which carrot pollen was being collected, given the sampling process used to make observations of carrot pollen counts. That is, the most likely rate of carrot pollen collection given the observed counts of carrot pollen during each sampling period, adjusted for the estimated total number of pollen balls collected during that period and the sub-sample of 60 balls used to determine the carrot pollen count.

Table S5. Number of pollen balls composed of carrot pollen with estimated number of pollen balls per sample.

i	date	time	mass	m100	est.	obs.c60
1	2004-01-06	12:00	7.81	0.67	1166	1
2	2004-01-06	17:30	10.04	0.80	1256	0
3	2004-01-12	12:00	7.67	0.68	1128	2
4	2004-01-12	21:30	3.66	0.78	470	4
5	2004-01-15	12:00	6.94	0.68	1022	2
6	2004-01-15	21:00	2.21	0.82	270	1
7	2004-01-19	12:00	4.40	0.64	688	1
8	2004-01-19	21:00	2.91	0.69	422	1
9	2004-01-23	12:00	6.38	0.55	1160	2
10	2004-01-23	21:00	3.85	0.58	664	2
11	2004-02-04	12:00	33.75	0.78	4328	0
12	2004-02-04	21:45	10.19	0.63	1618	0
13	2004-02-10	12:00	14.00	0.79	1772	0
14	2004-02-10	20:45	6.23	0.76	820	1
15	2004-02-13	12:00	34.87	0.76	4588	0
16	2004-02-13	20:45	2.52	0.56	450	2
17	2004-02-16	12:35	33.49	0.82	4084	0
18	2004-02-16	21:00	8.29	0.70	1184	0
19	2004-02-27	12:00	12.56	0.76	1654	0
20	2004-02-27	20:00	8.79	0.87	1010	0
21	2004-03-01	12:30	2.61	0.70	374	0

We begin by making the assumption that there is a "true" underlying rate of preference for carrot pollen μ, and that the sampling periods i = 1; 2; : : : ;N from which observations were obtained are in some sense representative of the larger set of hypothetical sampling periods from which we could potentially collect data. The individual probability of observing carrot pollen p_i in the ith sample period is related to the underlying mean as.

$$\mathrm{logit}\,(\rho_i) = \log\left(\frac{\rho_i}{1-\rho_i}\right) = b_i \sim N(\mu, \tau)$$

That is, the model includes sampling period as a random effect and the log-odds are normally distributed about the mean μ with precision τ .The actual model implemented in JAGS was as follows

```
model
{
    for (i in 1:N)
    {
       b[i] ~ dnorm(mu,tau)
       q[i] ~ dbin(p[i],n[i])
       r[i] ~ dbin(q[i]/n[i], 60)
       logit(p[i])  < - b[i]
    }
    pop.mean  < - exp(mu) / (1 + exp(mu))
    mu ~ dnorm(0.0,1.0E-6)
    sigma  < - 1 / sqrt(tau)
    tau ~ dgamma(0.001,0.001)
},
```

from which we can see that the process relies on a nested pair of binomial distributions. For each sampling period i = 1; 2; : : : ;N, the observed carrot pollen count r_i is used to generate a simulated count qi which adjusts for the sub-sample of size 60. We then use qi to estimate the probability pi of observing carrot pollen in a sample of size $\hat{n}_i$, the total estimated pollen ball count for period i. The quantity pi is related to the underlying population mean by equation (1), where μ is a measure expressed on the logit scale. For convenience we convert this back to the probability scale using

$$\hat{\theta} = \frac{\exp\ (\mu)}{1 + \exp(\mu)}$$

Where $\hat{\Theta}$ is the estimated underlying mean probability of observing carrot pollen in the population of sampling periods for which those considered here form a representative sample.

Results

Results from the model are provided in Table S6. Results from simulation model, where individual estimates for each sampling period i = 1; 2; : : : ; 21 are shown along with the estimates of the underlying mean, expressed as the log-odds μ and a probability. An indication of the precision of these estimates are provided by the posterior quantiles, of which interpretation is straightforward. The estimated mean probability of observing carrot pollen in

sample period 1 p1 (first day's sampling in morning, p2 afternoon of first day's sampling and so on) was 0.01488, or nearly 1.5 %, the median was 0.014, and 95 % of the samples generated against p1 lay in the interval [0:004503; 0:03052].

Table S6. Results from simulation model

param	mean	sd	2.5 %	50 %	97.5 %
μ	-4.32640	0.330232	-5.077342	-4.30163	-3.75547
P_1	0.01488	0.006872	0.004503	0.01400	0.03052
P_2	0.01293	0.005819	0.002268	0.01266	0.02570
P_3	0.01712	0.008161	0.006679	0.01545	0.03936
P_4	0.02194	0.014056	0.008461	0.01757	0.06294
p_5	0.01750	0.009097	0.006493	0.01565	0.04189
p_6	0.01520	0.006882	0.004430	0.01437	0.03142
p_7	0.01510	0.007314	0.004559	0.01413	0.03204
p_8	0.01494	0.007076	0.004394	0.01399	0.03163
p_9	0.01792	0.011209	0.006487	0.01556	0.04657
p_{10}	0.01777	0.010426	0.006339	0.01563	0.04478
p_{11}	0.01248	0.005807	0.001973	0.01246	0.02452
p_{12}	0.01344	0.006409	0.002201	0.01300	0.02757
p_{13}	0.01258	0.005762	0.002177	0.01233	0.02468
p_{14}	0.01495	0.006494	0.004407	0.01420	0.03048
p_{15}	0.01282	0.006001	0.002024	0.01248	0.02559
p_{16}	0.01763	0.009719	0.006445	0.01562	0.04328
p_{17}	0.01287	0.005726	0.001712	0.01263	0.02519
p_{18}	0.01300	0.006504	0.001611	0.01271	0.02653
p_{19}	0.01294	0.006102	0.002335	0.01258	0.02604
p_{20}	0.01326	0.005928	0.002628	0.01297	0.02602
p_{21}	0.01316	0.006357	0.002270	0.01282	0.02661
$\hat{\theta}$	0.01369	0.004107	0.006198	0.01337	0.02285

Discussion

The estimates of the individual sampling periods display the "shrinkage" characteristic of mixed-effects models (G. Lee pers, comm.). The estimates for the extreme observations are pulled in towards the overall mean. In the current scenario this is useful, because of the large number of zeroes in the data. However, the model also shows signs of instability due to the paucity of carrot pollen observations. If the core research question was to identify the underlying rate of pollen collection in the carrot crop by examination of the observed proportion of carrot pollen in the samples, it would have been useful to set the subsample count at a threshold which allowed a minimum carrot pollen count (in the range 5-10, say) for the majority of (and preferably all) sampling periods. This is recommended for any future study which aims to estimate this quantity with accuracy.

References

Bolker, B. M. (2008). Ecological Models and Data in R. Princeton University Press.

Lunn, D., Thomas, A., Best, N., and Spiegelhalter, D. (2000). WinBUGS - a Bayesian modelling framework: concepts, structure, and extensibility. Statistics and Computing, 10:325-337.

McCarthy, M. A. (2007). Bayesian Methods for Ecology. Cambridge University Press.

Plummer, M. (2003). JAGS: A program for analysis of bayesian graphical models using gibbs sampling. In Proceedings of the 3rd International Workshop on Distributed Statistical Computing, Vienna, Austria. ISSN 1609-395X.

Plummer, M., Best, N., Cowles, K., and Vines, K. (2009). coda: Output analysis and diagnostics for MCMC. R package version 0.13-4.

R Development Core Team (2010). R: A Language and Environment for Statistical Computing. R Foundation for Statistical Computing, Vienna, Austria. ISBN 3-900051-07-0.

Thomas, A., O`Hara, B., Ligges, U., and Sturtz., S. (2006). Making BUGS open. R News, 6:12-17.

Zuur, A. F., Ieno, E. N., Walker, N. J., Saveliev, A. A., and Smith, G. M. (2009). Mixed Effects Models and Extensions in Ecology with R. Springer.

3. Pollen morphology

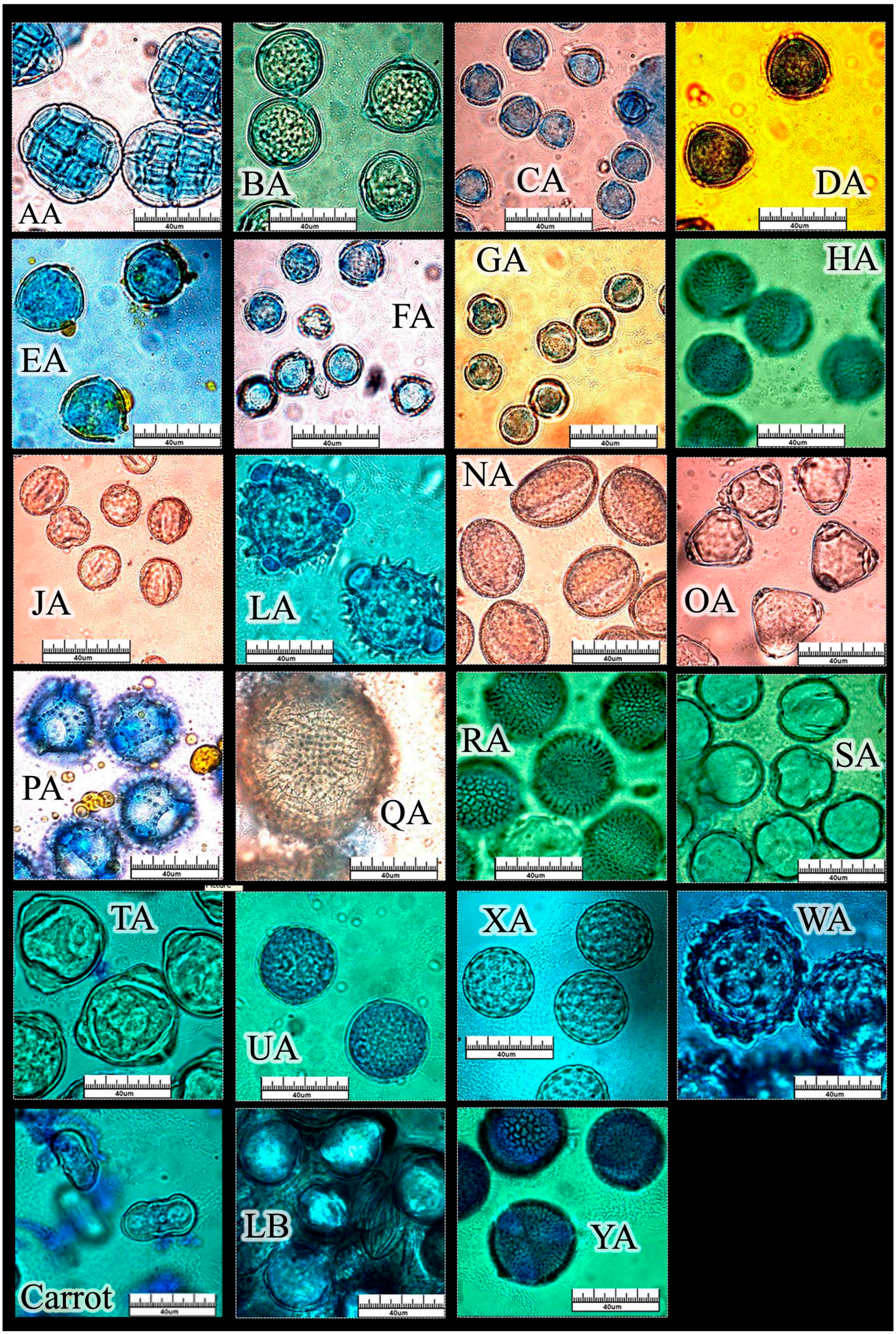

Figure S5. Pollen collected from the corbiculae of honey bees in hives adjacent to a hive pollen trap adjacent to hybrid carrot crops in 2003/04. (AA = Acacia, GA = *Bursaria spinosa*, XA = Chenopodiaceae, WA = Malvaceae,.QA = Euphorbiaceae, PA = Asteraceae, QA = Myrtaceae, SA = Dilleniaceae). Carrot pollen is shown in the bottom left-hand corner labelled 'carrot'.
